# The Effects of Glucosinolates and Their Breakdown Products on Necrotrophic Fungi

**DOI:** 10.1371/journal.pone.0070771

**Published:** 2013-08-05

**Authors:** Kobi Buxdorf, Hila Yaffe, Omer Barda, Maggie Levy

**Affiliations:** Department of Plant Pathology and Microbiology, The Robert H. Smith Faculty of Agriculture, Food and Environment, The Hebrew University of Jerusalem, Rehovot, Israel; Portland State University, United States of America

## Abstract

Glucosinolates are a diverse class of S- and N-containing secondary metabolites that play a variety of roles in plant defense. In this study, we used *Arabidopsis thaliana* mutants that contain different amounts of glucosinolates and glucosinolate-breakdown products to study the effects of these phytochemicals on phytopathogenic fungi. We compared the fungus *Botrytis cinerea*, which infects a variety of hosts, with the Brassicaceae-specific fungus *Alternaria brassicicola*. *B. cinerea* isolates showed variable composition-dependent sensitivity to glucosinolates and their hydrolysis products, while *A. brassicicola* was more strongly affected by aliphatic glucosinolates and isothiocyanates as decomposition products. We also found that *B. cinerea* stimulates the accumulation of glucosinolates to a greater extent than *A. brassicicola*. In our work with *A. brassicicola*, we found that the type of glucosinolate-breakdown product is more important than the type of glucosinolate from which that product was derived, as demonstrated by the sensitivity of the Ler background and the sensitivity gained in Col-0 plants expressing epithiospecifier protein both of which accumulate simple nitrile and epithionitriles, but not isothiocyanates. Furthermore, *in vivo*, hydrolysis products of indole glucosinolates were found to be involved in defense against *B. cinerea*, but not in the host response to *A. brassicicola*. We suggest that the Brassicaceae-specialist *A. brassicicola* has adapted to the presence of indolic glucosinolates and can cope with their hydrolysis products. In contrast, some isolates of the generalist *B. cinerea* are more sensitive to these phytochemicals.

## Introduction

Glucosinolates are a diverse class of S- and N-containing secondary metabolites that are found mainly in members of the Brassicaceae [1]. GSs Glucosinolates play a variety of roles in plant defense responses and cancer prevention. They are relatively nonreactive, hydrophilic, nonvolatile compounds that are stored within plant vacuoles [2], [3]. Significant progress has been made in understanding the biochemistry and genetics of glucosinolates biosynthesis [4], [5] and how that biosynthesis is regulated over the course of plant development and in response to environmental cues [6], [7].

**Figure 1:**
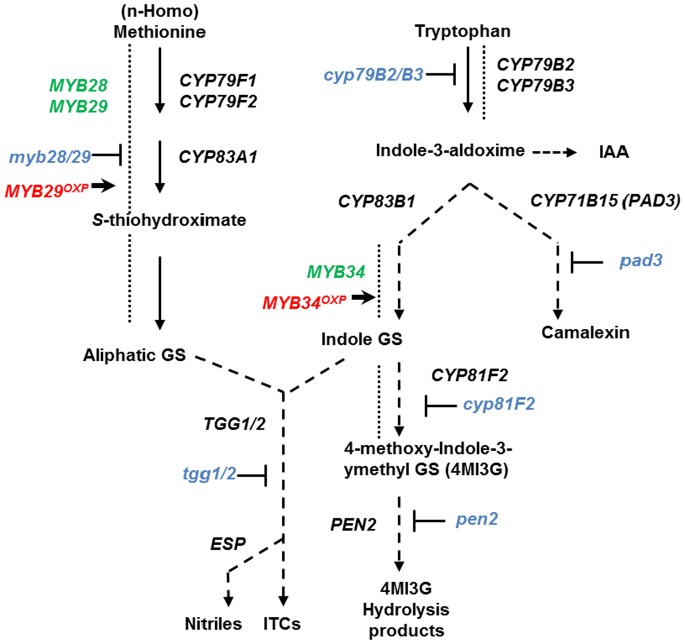
Biosynthesis of glucosinolates and camalexin. Schematic overview of genes (black letters) and mutations (blue letters) implicated in the biosynthesis of camalexin and the biosynthesis and breakdown of glucosinolates. The transcription factors (green letters) *MYB28* and *MYB29* regulate the expression of genes involved in the aliphatic gucosinolate pathway and *MYB34* regulates the expression of genes involved in the indolic glucosinolate pathway.

They hydrolysis of glucosinolates is catalyzed by endogenous myrosinases (β-thioglucoside glucohydrolases) [8]. Myrosinases are encoded by small gene family and are found in idioblasts [9] in most tissues of glucosinolate-producing plants [10], [11]. Upon plant injury, glucosinolates are rapidly hydrolyzed by myrosinases into a multitude of physiologically active products, including isothiocyanates (ITCs), thiocyanates, simple nitriles, epithionitriles and oxazolidine-2-thiones [11]–[15]. The chemical structure of the side chain of intact glucosinolate, the presence of myrosinase-associated or specifier proteins and other environmental factors, such as pH and the presence of metal ions, may affect the types of hydrolysis products formed [4]. Myrosinase-associated proteins include the *Arabidopsis thaliana* epithiospecifier protein (ESP) modifier 1 (ESM1) [16]. Specifier proteins include nitrile-forming proteins, such as the ESP from *A. thaliana* that responsible for epithionitriles formation (Figure 1) [15], [17] and the nitrile-specifier proteins (NSPs) that promote the formation of simple nitriles [14], [15].

The glucosinolate-myrosinase system can be considered a binary, spatially separated chemical defense system that is activated upon tissue disruption. It has been proposed that glucosinolate-breakdown products may participate in plant defense responses to herbivores, nematodes and pathogens [18]–[24].

A negative correlation or the lack of a correlation between glucosinolate content and resistance to the *Brassica-*specialist herbivores and pathogens has been reported in different pathosystems [25]–[28]. Those reports suggest that specialist pests may have evolved various mechanisms that take advantage of the production of specific glucosinolates in particular plants. Indeed, herbivores specializing on glucosinolate-containing plants have found different ways to adapt to the presence of glucosinolates or overcome glucosinolate-breakdown products’ toxicity [13], [29]. Recent studies have shown that different areas of the leaf accumulate different concentrations of glucosinolates within their cells and thus may differ in their response to insects [30]–[32].

Together with camalexin, glucosinolates have been shown to play a role in plant defense against fungal pathogens. Mutant plant lines deficient in camalexin or in indolic or aliphatic glucosinolate biosynthesis were hypersusceptible to *Sclerotinia sclerotium* [33]. Inoculation of *Arabidopsis* plants with adapted or non-adapted isolates of the ascomycete *Plectosphaerella cucumerina* triggered the accumulation of indolic glucosinolates, which were reported to play a key role in limiting the growth of both non-adapted and adapted necrotrophic fungi but not of the non-adapted biotrophic fungus *Erysiphe pisi* [34]. The *Arabidopsis* indole glucosinolate pathway has also been found to restrict entry of a non-adapted *Colletotricum* sp. anthracnose fungus [35]. Schlaepii *et al.* (2010) [36] demonstrated that both camalexin and products of indolic glucosinolate hydrolysis are important for disease resistance to *Phytophthora brassicae*. Furthermore, *Fusarium oxysporum* was reported to be significantly more aggressive on *gsm1-1*, a mutant deficient in aliphatic glucosinolate than on wild-type plants [24].

Indolic glucosinolates have recently been shown to be essential for defense against pathogens and to mediate innate immunity [34], [36]–[38]. The indolic glucosinolates defense pathway involves the cytochrome P450 monooxygenase CYP81F2, which is essential for pathogen-induced accumulation of 4-methoxyindol-3-ylmethylglucosinolate (4MI3G). 4MI3G can be hydrolyzed by the Penetration 2 (PEN2) myrosinase into metabolites that are involved in defense against fungal pathogens (**Figure 1**) [37]. PEN2 and CYP81F2 are involved in plant defense responses to a variety of pathogens, including the fungal pathogens *Plectosphaerella cucumerina* [34] and *Colletotricum* sp. [35]and oomycetes such as *Phytophthora brassicae* [36] and *Pythium irregulare* [39].

We studied the effect that glucosinolates have on the broad-spectrum pathogen *Botrytis cinerea* and the specialist *Brassica* pathogen *Alternaria brassicicola*. Using *A. thaliana* mutants whose glucosinolate contents had been altered, we showed that *B. cinerea* displayed variable sensitivity to glucosinolates and their degradation products; whereas *A. brassicicola* was more tolerant of glucosinolates and their hydrolysis products. We also discovered that for *A. brassicicola* the effect of the type of glucosinolate-breakdown product is stronger than the effect of the glucosinolates group from which the glucosinolate-breakdown product was derived. We demonstrated that the hydrolysis products of indolic glucosinolates are responsible for the differences observed between plant responses to *B. cinerea* and plant responses to *A. brassicicola.*

## Materials and Methods

### Plant Lines and Growth Conditions

This work was carried out using the following *Arabidopsis thaliana* (L.) Heynh. accessions: Col-0, Ws-0 and Ler; and the following mutants and transgenic plants: *tgg1-3/tgg2-1* [23](Col-0); *35S:ESP* [40](Col-0); *cyp79B2*, *cyp79B3* and *cyp79B2/79B3* [41](Col-0 and Ws-0); *Myb34^OXP^* and *Myb29^OXP^* [42](Ler); *tgg1tgg2∶35S:ESP* [43](Col-0); *pad3* [44](Col-0); *pen2-2*, *cyp81F2* and *pen2/cyp81F2* [37](Col-0). All seeds were scarified on moist soil at 4°C for 2 to 3 days before they were placed in a growth chamber. Plants were grown at 22°C and 60% relative humidity under fluorescent and incandescent light at a photofluency rate of approximately 120 µmol m^−2^ s^−1^ and a 12/12 h photoperiod.

### Fungal Strains, Growth and Inoculation Method

*B. cinerea* strain B05.10 (sequenced isolate obtained from Syngenta) [45]) an isolate of *B. cinerea* isolated from *Vitis vinifera*, which we will refer to as the grape isolate, and *A. brassicicola* (isolated in from infected *Brassica oleracea* var. *capitata*) were grown on potato dextrose agar (PDA; Difco, France) in a controlled-environment chamber kept at 22°C under fluorescent and incandescent light at a photofluency rate of approximately 120 µmol m^−2^ s^−1^ and a 12/12 h photoperiod. Conidia were harvested in sterile, distilled water and filtered through four layers of sterile gauze to remove any clinging hyphae. For inoculation, the conidial suspension was adjusted to 3000 conidia µl^−1^. The *B. cinerea* conidial suspension was prepared in half-strength filtered (0.45 µM) grape juice (100% organic grape juice) and the *A. brassicicola* conidial suspension was prepared in water. Detached leaves from the different genotypes were layered on trays of water-agar media and inoculated with 5-µl droplets of conidial suspension. Since different areas of the leaf accumulate different concentration of glucosinolates within their cells and they may differ in their responses to fungal inoculation [30]–[32], we selected the leaf’s main vain as the preferred inoculation site. Lesions were measured using ASSESS 2.0, image analysis software for plant disease quantification (APS Press, St. Paul, MN, USA). All data presented are representative of at least three independent experiments with similar results.

### HPLC Analysis of Desulfoglucosinolates

Glucosinolates were extracted from whole leaves of 3-week-old *A. thaliana* plants (100 mg fresh weight). The leaves were boiled in 1 ml dd H_2_O, the broth was collected and the leaves were then washed with another 1 ml dd H_2_O. The combined broth and washing fluid (2 ml) was applied to a DEAE-Sephadex A-25 (40 mg) column (pyridine acetate form). To convert the glucosinolates into their desulfo analogs, we treated them overnight with 100 µl 0.1% (1.4 units) aryl sulfatase (Sigma-Aldrich). The desulfoglucosinolates were then eluted with 1 ml dd H_2_O. HPLC of desulfoglucosinolates was carried out using an Agilent Technologies 1200 Liquid Chromatograph. Samples (100 µl each) were separated at ambient temperature on an EKA KR100-5C18 column (250 × 4.6 mm i.d., 5-µm particle size), using the acetonitrile gradient described below in dd H_2_O at a flow rate of 1.0 ml min^−1^. The column was developed by isocratic elution with 1.5% acetonitrile (5 min), followed by a linear gradient to 20% acetonitrile (15 min) and isocratic elution with 20% acetonitrile (10 min). Absorbance was detected at 226 and 280 nm. Desulfoglucosinolate concentrations were calculated based on published response factors developed using sinigrin (allyl glucosinolate) as a standard [46], [47].

### Statistical Analysis

A *t*-test was performed only when data were normally distributed and samples had equal variances. In all other cases, a Mann-Whitney Rank Sum Test was performed. For multiple comparisons, one-way ANOVA analysis was performed when the data passed the equal variance test. In all other cases, one-way ANOVA analysis on ranks was performed (Kruskal-Wallis). For multiple factors, Dunn’s test was performed. Differences were considered to be significant at *P*<0.05.

## Results

### *Alternaria brassicicola* is More Strongly Affected by Aliphatic Glucosinolates and Camalexin than by Indolic Glucosinolates

*Arabidopsis* plants contain mainly methionine-derived (aliphatic) or tryptophan-derived (indolic) glucosinolates [47], [48] (Figure 1). *A. thaliana* ecotypes that contain different mixtures of glucosinolates [47], [48]were inoculated with *B. cinerea* isolated from infected *Vitis vinifera* (grape isolate), the *B. cinerea* isolate B05.10 (whose genome has been sequenced), or *A. brassicicola* isolated from infected *Brassica oleracea* var. *capitata* (cabbage) grown in southern Israel. The different *A. thaliana* wild-type ecotypes demonstrated differential susceptibility to these pathogens (**Figure S1**).

**Figure 2:**
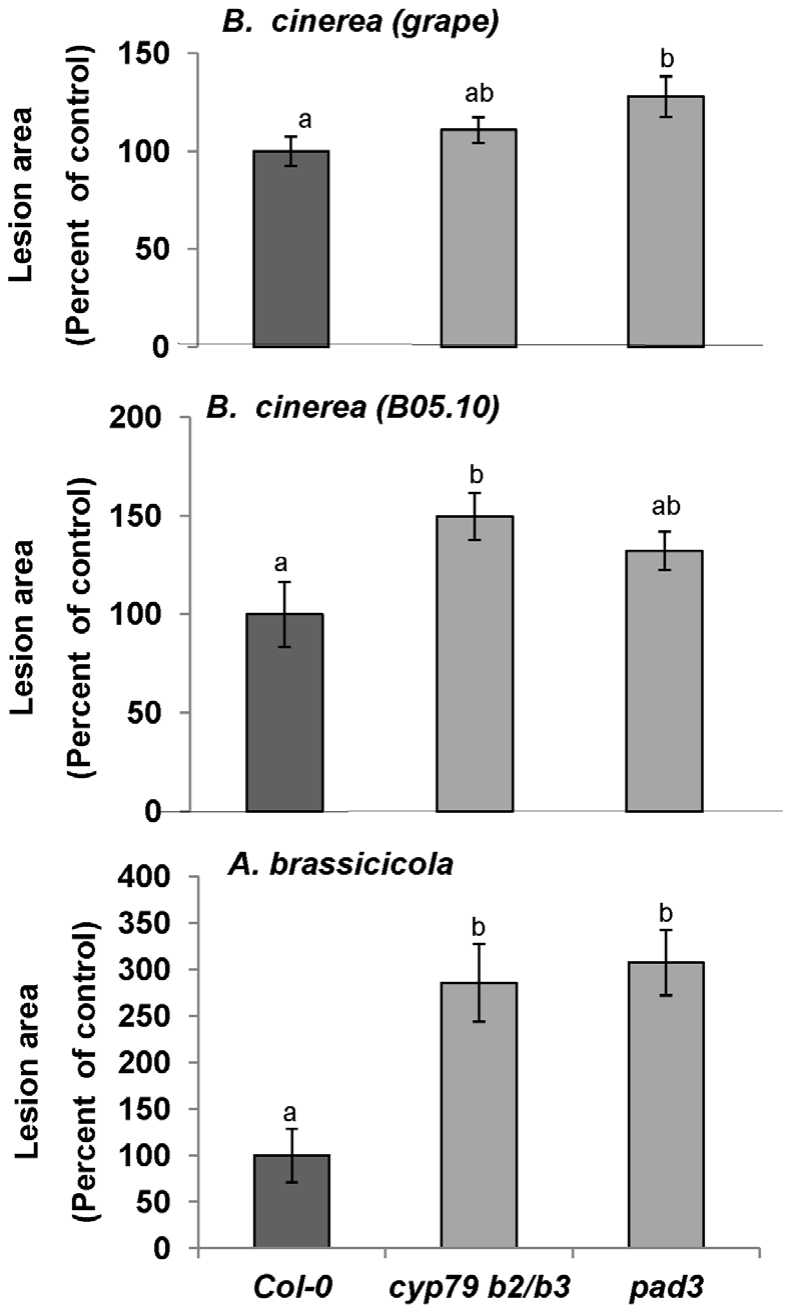
Effects of indole glucosinolate and camalexin on fungal pathogenicity. *Arabidopsis* mutants *cyp79B2/B3* and *pad3*, which have altered total glucosinolate and/or camalexin content, and their corresponding wild-type background (Col-0) were inoculated with *B. cinerea* (B05.10 or grape isolate) or *A. brassicicola*. Lesion size was measured 72 h after inoculation (upper and middle panels) with *B. cinerea* and 120 to 192 h after inoculation with *A. brassicicola* (lower panel). Average lesion sizes from 30 leaves of each genotype are presented along with and the standard error of each average. All numbers are presented as the relative percentage to their corresponding background wild-type. Different letters above the columns indicate statistically significant differences at *P*<0.05, as determined using the Kruskal-Wallis test and Dunn’s test.

To determine whether indolic glucosinolates affect fungal pathogenesis we used the *Arabidopsis cyp79B2 cyp79B3 (cyp79B2/B3*) double mutant with a Col-0 background, which does not accumulate indolic glucosinolates or camalexin and whose aliphatic glucosinolates levels are 50% or less than the aliphatic glucosinolates levels observed in the wild-type [49], [50]. This double mutant exhibited enhanced sensitivity to *A. brassicicola* (Figure 2, lower panel), and a moderately higher (although not always significant so) levels of sensitivity to the B05.10 *B. cinerea* isolate and to the grape isolate as compared with wild-type plants (Figure 2, upper and middle panels). In the plants in which this mutation was expressed against the Ws-0 background, the same pattern of resistance was observed for interactions with *A. brassicicola* and the B05.10 isolate of *B. cinerea* (Figure S2). Since the *cyp79B2/B3* double mutant also has impaired camalexin accumulation [41], we compared its sensitivity to that of the camalexin-deficient mutant *pad3* [44], [51]. As shown in Figure 2, *pad3* plants were more sensitive than the wild-type to *A. brassicicola* and *B. cinerea*. However, the sensitivities of the *pad3* plants did not differ from those of the *cyp79B2/B3* mutant.

**Figure 3:**
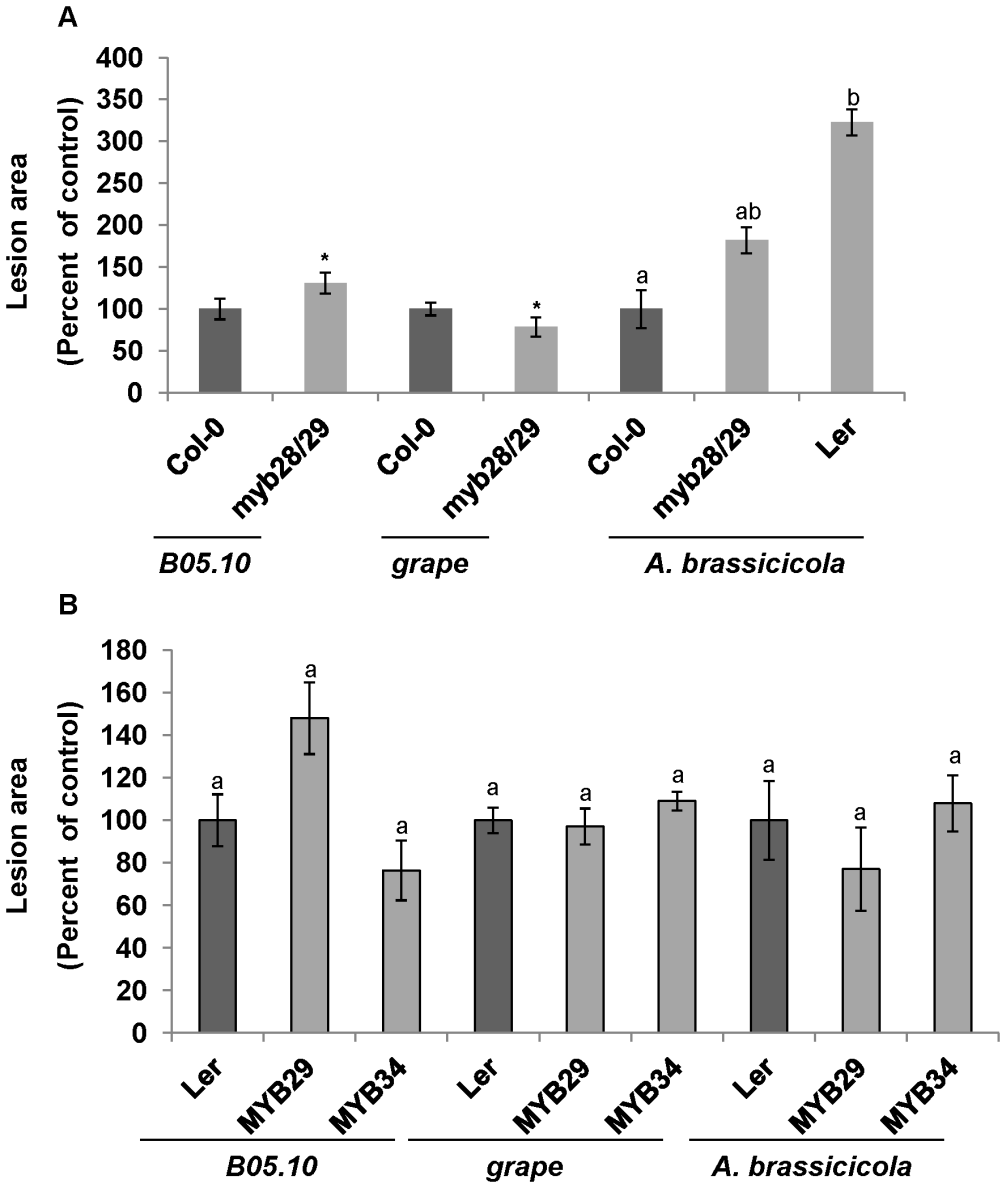
Impact of aliphatic glucosinolate on fungal pathogenicity. *Arabidopsis* leaves from plants containing the double-knockout *myb28 myb29* (*myb28/29*) expressed against the Col-0 background (A) and plants in which *MYB29^OXP^* (MYB29) and *MYB34^OXP^* (MYB34) were expressed against the Ler background (B) were inoculated with *B. cinerea* (B05.10 or grape isolate) or *A. brassicicola*. Lesion size was measured 72 h after inoculation with *B. cinerea* and 120 to 192 h after inoculation with *A. brassicicola*. Average lesion sizes from 10 to 17 leaves of each genotype are presented together with the standard errors for each average. All numbers are presented as the relative lesion size as compared to that observed on the corresponding background wild-type plants. Different letters or asterisks above the columns indicate statistically significant differences at *P*>0.05, as determined using the Kruskal-Wallis test and Dunn’s test.

We also examined whether aliphatic glucosinolates affect fungal pathogenesis. We used an *Arabidopsis* double mutant *myb28 myb29* (*myb28/29*) that lacks aliphatic glucosinolates [33], [52]–[56]and has the Col-0 background. This mutant was significantly more susceptible to the B05.10 *B. cinerea* isolate and significantly more resistant to the grape isolate of *B. cinerea*. Its level of sensitivity to *A. brassicicola* was between that of the naturally resistant Col-0 ecotype and that of the sensitive Ler ecotype (**Figure 3**A). In addition we used two *Arabidopsis* mutants that had the Ler background. The first mutant overexpresses the MYB29 transcription factor (*MYB29^OXP^*) and contains high levels of aliphatic glucosinolates and low levels of indolic glucosinolates and camalexin. The second mutant overexpresses the MYB34 transcription factor (*MYB34^OXP^*) and contains high levels of indolic glucosinolates and camalexin and low levels of aliphatic glucosinolates [42]. Camalexin levels in these mutants were determined after treatment with AgNO_3_, not after pathogen infection. We found no significant differences in the sensitivity of these two mutants to the *B. cinerea* isolates and *A. brassicicola* (Figure 3B). Overall, our data suggest that *A. brassicicola* is more sensitive to camalexin than *B. cinerea.* Moreover, *A*. *brassicicola* and the B05.10 *B. cinerea* isolate are more sensitive to aliphatic glucosinolates than the grape isolate of *B. cinerea*. These differences may be attributed, in part, to the type of aliphatic glucosinolates present in Ler and Col-0 and to the notion that glucosinolate breakdown in Ler or Col-0 results mostly in nitrile or ITC production, respectively [47], [48].

### Indolic Glucosinolate Turnover Products may be involved in Defense against *B. cinerea*

**Figure 4:**
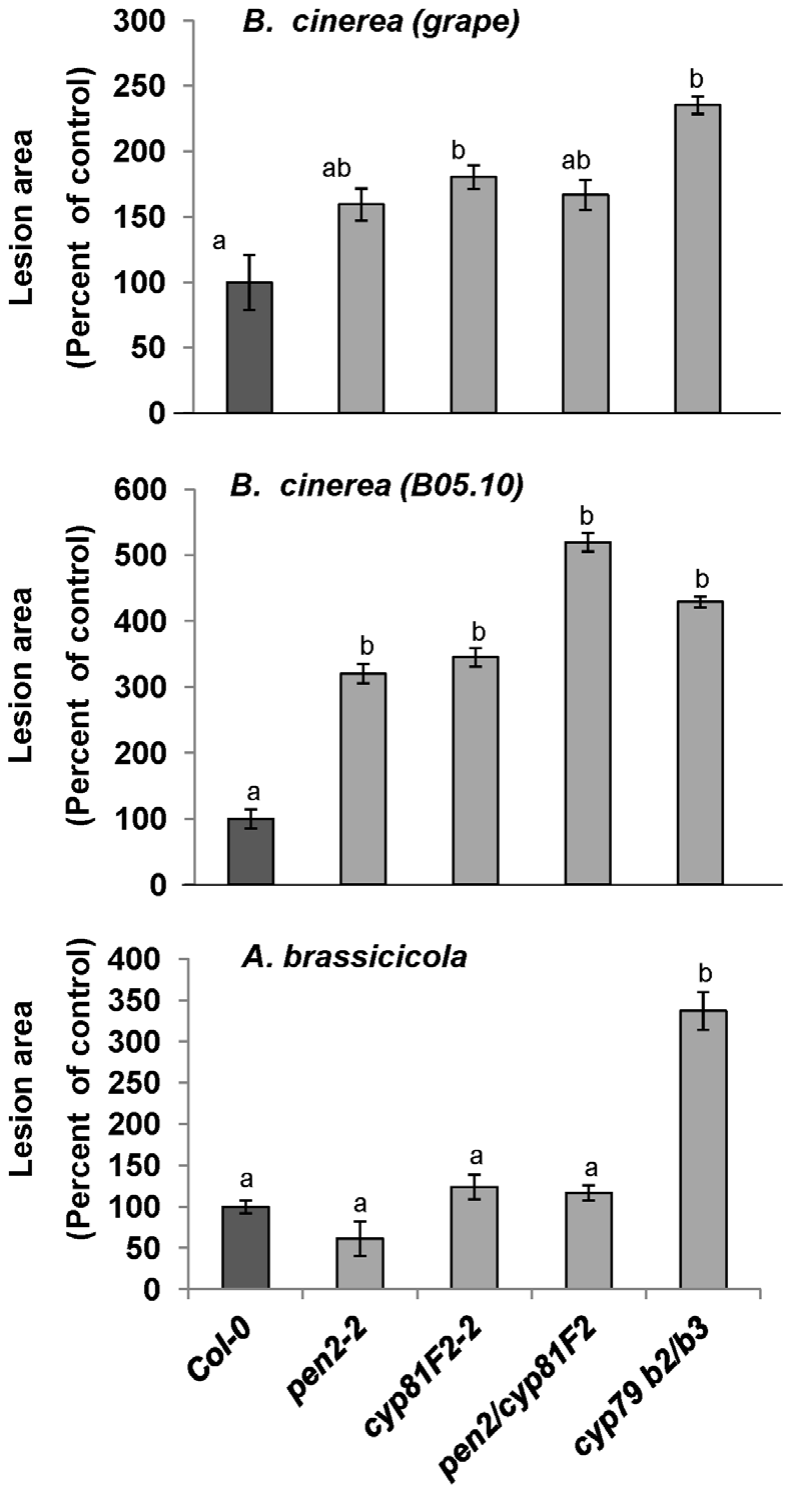
Effects of glucosinolate-turnover products on fungal pathogenicity. *Arabidopsis* leaves from wild-type, *pen2*, *cyp81F2* and *pen2/cyp81F2* plants were inoculated with the grape isolate of *B. cinerea* (upper panel), the B05.10 *B. cinerea* isolate (middle panel) or *A. brassicicola* (lower panel). Lesion size was measured 72 h after inoculation with *B. cinerea* and 120 to 192 h after inoculation with *A. brassicicola*. Average lesion areas for 30 leaves of each genotype are presented together with the standard error for each average. All numbers are presented as the relative lesion size as compared to the lesions observed on the corresponding background wild-type plants. Different letters above the columns indicate statistically significant differences at *P*<0.05, as determined using the Kruskal-Wallis test and Dunn’s test.

A novel glucosinolate metabolic pathway has recently been revealed, which differs from the pathway activated by insects [37], [38]. This pathway involves *CYP81F2* and *PEN2*, which have myrosinase activity associated with defense responses against hemi-biothrophic, biotrophic and adapted necrotrophic fungal pathogens [34], [37], [57]. When we examined *pen2-2* and *cyp81F2-2* mutants and the *pen2/cyp81F2* double mutant, we found no difference in the resistance of plants with these mutations to *A. brassicicola* relative to their corresponding background, wild-type Col-0 (Figure 4, lower panel). In contrast, plants containing the *cyp81F2-2* mutation were more sensitive to both *B. cinerea* isolates and *pen2-2* was more sensitive to the B05.10 isolate (compare Figure 4, upper and middle panels). The *pen2/cyp81F2* double mutant exhibited increased sensitivity only to the B05.10 *B. cinerea* isolate. The sensitivity of the *pen2-2* and *cyp81F2* mutants and of the *pen2/cyp81F2* double mutant to both *B. cinerea* isolates was comparable to the sensitivity of the *cyp79B2/B3* plants (compare Figure 4, upper and middle panels). These results indicate that indolic glucosinolate-turnover products may be involved in defense against *B. cinerea*, but not in defense against *A. brassicicola*.

### *A. brassicicola* is more Sensitive to Isothiocyanates than Epithioitriles

**Figure 5:**
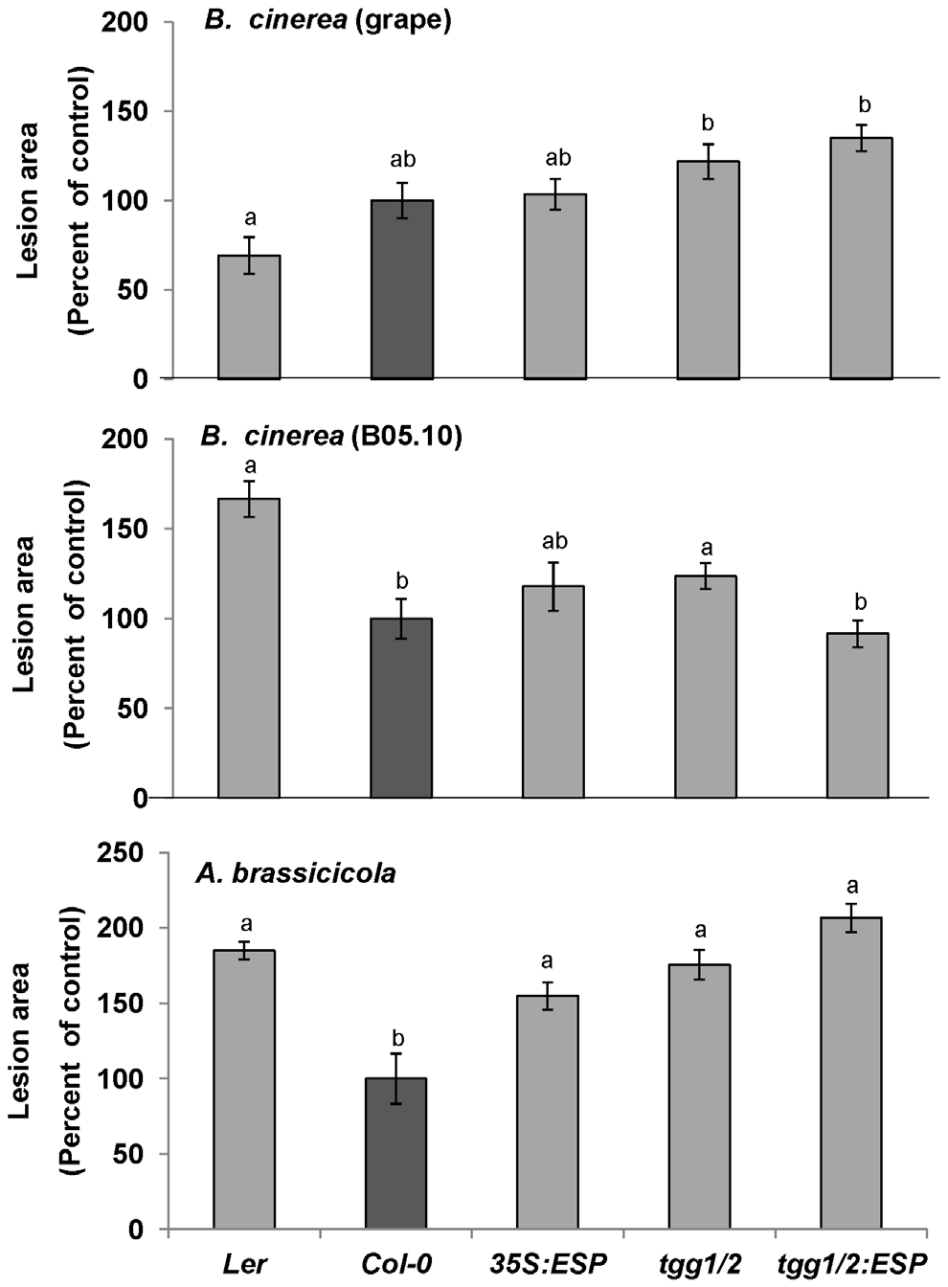
Effects of glucosinolate-breakdown products on fungal pathogenicity. *Arabidopsis* mutants with altered total glucosinolate-breakdown product contents and containing different relative amounts of the different type of products were inoculated with the grape isolate of *B. cinerea* (upper panel), the B05.10 isolate of *B. cinerea* (middle panel) or *A. brassicicola* (lower panel). Lesion size was measured 72 h or 120 to 192 h post-inoculation (*B. cinerea* and *A. brassicicola*, respectively) on leaves from *tgg1-3/tgg2-1* (*tgg1/2*) plants, *35S:ESP* plants, the wild-types Col-0 and Ler and the triple mutant *35:ESP/tgg1-3/tgg2-1* (*tgg1/2:ESP*). (All mutations were expressed against the Col-0 background.) Average lesion areas from 15 to 30 leaves of each genotype are presented together with the standard error of each average. All numbers are presented as the relative lesion size as compared to that observed on the corresponding background wild-type plants. Different letters above the columns indicate statistically significant differences at *P*<0.05, as determined using the Kruskal-Wallis test and Dunn’s test.

To examine the effects of glucosinolate-breakdown products on fungal pathogenesis, we used the *tgg1-3/tgg2-1* (*tgg1/2*) double-knockout mutant lacking myrosinase activity [23]. Analysis of the pathogens’ virulence toward the *tgg1/2* mutant demonstrated that *A. brassicicola* and the B05.10 *B. cinerea* isolate are sensitive to glucosinolate-breakdown products; whereas the grape isolate of *B. cinerea* is less sensitive (**Figure 5**). Furthermore, in experiments carried out using mutants that overexpress the root myrosinase TGG4, we observed no significant differences in the pathogenicity of the different fungi (Figure S3). This suggests that *A. brassicicola* and the B05.10 *B. cinerea* isolate are sensitive to *TGG1/TGG2-*derived glucosinolate-breakdown products; whereas the grape isolate of *B. cinerea* has the ability to detoxify or tolerate these glucosinolate-breakdown products.

We analyzed the *in vitro* antifungal activity of different glucosinolates-derived ITCs against *B. cinerea* isolates and *A. brassicicola*. While *A. brassicicola* was affected by most ITCs (Figure S5 and Data S1), *B. cinerea* was more resistant (Figure S4 and Data S1).

To verify which class of glucosinolate-breakdown products has a stronger effect on these fungi, we performed a pathogenicity analysis on wild-type Col-0 plants. Most of the glucosinolate-breakdown products found in these plants are ITCs and simple nitriles, due to the inactive ESP protein in these plants [17], [58]. We also examined transgenic plants with the Col-0 background that overexpress ESP under the control of a 35S promoter (*35S:ESP*) and in which, like in the Ler wild-type, simple and epithionitriles account for most of the glucosinolate-breakdown products ([59]. We found no differences in resistance when these plants were inoculated with the *B. cinerea* isolates; however, the *35S:ESP* plants were as sensitive to *A. brassicicola* as the Ler wild-type plants (Figure 5). Furthermore, inoculation assays in which the *tgg1/2*::35S*:ESP (tgg1/2:ESP)* triple mutant that was compared with its Col-0 background and with the *tgg1/2* double mutant and Ler wild-type demonstrated that these mutations do not affect plant response to inoculation with *A. brassicicola* or the grape isolate of *B. cinerea* (Figure 5). Taken together, these results indicate that *A. brassicicola* is more sensitive to ITCs than to epithionitriles; whereas *B. cinerea* has similar sensitivity/resistance to both types of glucosinolate-breakdown products.

### *B. cinerea* B05.10 Induces Glucosinolate Accumulation more Strongly than *A. brassicicola*

**Figure 6:**
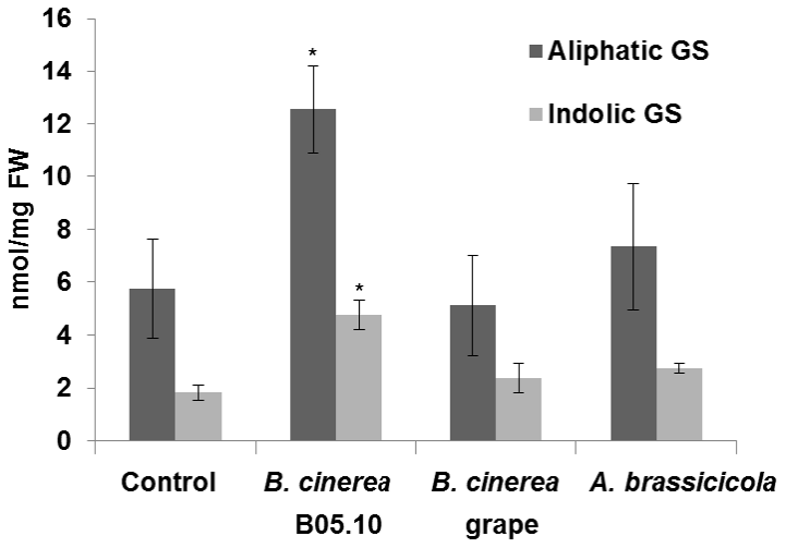
Glucosinolate accumulation in *Arabidopsis* after inoculation with a fungal pathogen. Col-0 *Arabidopsis* seedlings were inoculated with the B05.10 *B. cinerea* isolate or *A. brassicicola* and glucosinolate content was measured 72 h or 120 to 192 h post-inoculation, respectively. GS, glucosinolate. Average glucosinolate accumulation was calculated for 6 to 9 seedlings per treatment and those averages are presented together with their standard errors. Asterisks indicate statistically significant differences relative to the control at *P*<0.05, as indicated by *t*-tests.

Although glucosinolates are preformed secondary metabolites (phytoanticipins), the amounts of these compounds can change following a variety of stimuli [60]–[62], as well as following exposure to insects and pathogens [36], [63], [64]. HPLC glucosinolate analysis was performed on *Arabidopsis* plants that had been inoculated with either *B. cinerea* or *A. brassicicola*. This analysis revealed that plants inoculated with the B05.10 isolate of *B. cinerea* accumulated two-fold more glucosinolate than uninoculated plants. On the other hand, inoculation with *A. brassicicola* or the grape isolate of *B. cinerea* did not affect glucosinolate accumulation or profile (Figure 6). Thus, it appears that the B05.10 isolate of *B. cinerea* stimulates glucosinolate accumulation to a greater extent than *A. brassicicola*.

## Discussion

We characterized the effects of glucosinolates and their hydrolysis products on two different isolates of the necrotrophic fungus *B. cinerea*, a broad-spectrum pathogen, and on the crucifer-specialized necrotrophic fungus *A. brassicicola*. While both pathogens have a similar lifestyle, we demonstrated the differential effects of glucosinolates and their breakdown products on the pathogenicity of these two fungi.

It is well established that different *Arabidopsis* ecotypes differ in their glucosinolate profiles [48]. We observed differences among the ecotypes we tested with respect to *A. brassicicola* resistance/sensitivity (**Figure S1**). We, therefore, wanted to determine whether, among other differences in defense response obtained in the different ecotypes, glucosinolate content is also important for the differences in plant sensitivity and resistance to the fungi used in this study. Careful examination of Arabidopsis mutants revealed that aliphatic glucosinolate may have a stronger effect on *A. brassicicola* than indolic glucosinolates, whereas the effects of these compounds on *B. cinerea* were isolate-dependent (**Figures 2** and **3**). Differential sensitivity to glucosinolate levels among different *B. cinerea* isolates has also been reported by Kliebenstein et al. (2005) [49].

Following the inoculation of the *myb28/29* double mutant, which does not accumulate aliphatic glucosinolates [52], we observed that this set of mutations has a negative effect on the plant’s ability to defend itself against *A. brassicicola* and *B. cinerea* isolate B05.10. This is similar to findings reported for *Sclerotinia sclerotiorum*, a close relative of *B. cinerea* [33]. We also observed a minor significant positive effect of this set of mutations on these plants’ ability to defend themselves against the grape *B. cinerea* isolate (20% decrease in lesion size; **Figure 3**). The *myb28/29* mutation might also affect the accumulation of secondary metabolites other than aliphatic glucosinolates, as Malitskey et al. (2008) [42]suggested for MYB29^OXP^ plants. Those additional effects might explain the negative effect of these mutations on the virulence of the grape isolate. Alternatively, aliphatic glucosinolates might have a direct, positive effect on the virulence of this isolate, as reported for *Pseudomonas* species [56].

When indolic glucosinolates and camalexin are absent, as in the case of the *cyp79B2/B3* double mutant, Col-0 plants, which are naturally resistant to *A. brassicicola*, and Ws-0 plants, which are slightly less resistant, both become very sensitive to the pathogen, showing over 200% increase in lesion size as compare to wild-type(**Figures 2** **,** **4** and **S2**). The fact that the sensitivity of the *cyp79B2/B3* mutant to *A. brassicicola* was comparable to that of the *pad3* mutant (**Figure 2**) suggests that camalexin, not indolic glucosinolates may play the main role in resistance to this fungus.

Our data suggest that other secondary metabolites may also be involved in the examined plant-pathogen interactions. For example, the *cyp79B2/B3* double mutant also has altered production of indole-3-carboxylic acids [65]. The production of these compounds is induced in *A. thaliana* following fungal infection and their absence may compromise defense responses [66]–[68]. Moreover, the accumulation of a broad spectrum of secondary metabolites and/or other immunity factors in the MYB mutants that we used in this study might differ from the accumulation of these substances in wild-type plants [42]. Another possible scenario might involve the redirection of secondary metabolism from camalexin to glucosinolates (or the reverse) by the fungi, as was recently reported following activation of the *Arabidopsis* gene miR393 [69].

As demonstrated in a publicly available microarray analysis (https://www.genevestigator.com/gv/index.jsp), a plant activates part of its indolic glucosinolates pathway (i.e., *CYP79B2* and *MYB29*) following infection by *A. brassicicola* (**Table S1**). On the other hand, *MYB51* and *PEN2*-dependent hydrolysis of the indolic glucosinolates pathway is downregulated following *A. brassicicola* infection. We suggest that these changes in gene expression may derive from fungal manipulation of the plant’s defense responses, in order to avoid toxic/signaling compounds. Indeed, in line with our findings, *PEN2*-generated decomposition products do not contribute to Col-0’s resistance to *A. brassicicola*. This is probably because *A. brassicicola* decreases the accumulation of those products in the plant (**Figure 4** and **Table S1**). Alternatively, the fungus may be resistant to those compounds, or the plant cells might fail to respond due to lack of fungus recognition. The expression profile of glucosinolate-related genes following *A. brassicicola* infection might reflect negative interactions between tryptophan- and methionine-derived glucosinolates and might only be valid for the specific time points examined [42], [70]. Genes from the aliphatic glucosinolates pathway were also activated to some extent following *A. brassicicola* infection (**Table S1**); this did not correlate with our findings demonstrating that the accumulation of both types of glucosinolates following *A. brassicicola* infection was almost not affected (**Figure 6**). Never the less, probably due to differences in the pathosystems, the accumulation of indolic glucosinolates has been observed following *Alternaria brassicae* infection and herbivory by the specialist *Phaedon cochleariae* [63], as well as infection with *Phytophthora brassicae* [36].

In contrast, the generalist *B. cinerea* has less of an effect on the activation of genes in the glucosinolate regulation, turnover and degradation pathways (Table S1) and stronger effects on the expression of genes involved in tryptophan and indolic glucosinolates biosynthesis as compared to *A. brassicicola*, which is significantly correlated with greater glucosinolate accumulation after infection by the B05.10 isolate (**Table S1 and** **Figure 6**). Nevertheless, according to the publicly available microarray data, aliphatic glucosinolates accumulation is not correlated with gene activation. This difference between our findings and the public microarray data may be due to differences in experimental procedures. It is important to note that we demonstrated a large variety of glucosinolate effects on the different *B. cinerea* isolates and that this variation may also apply to gene activation.

Glucosinolates are relatively nonreactive, but their breakdown products have strong effects on different insects and pathogens [24], [58], [71], [72]. Our data show that the identity of glucosinolate-breakdown product is important for the pathogenicity of *A. brassicicola*, but not for that of *B. cinerea*. When the glucosinolate-breakdown product mixtures were shifted from an ITCs to epithionitriles via expression of functional ESP against the Col-0 background, plants became sensitive to *A. brassicicola* (**Figure 5**). This suggests that the effect of glucosinolate-breakdown product type (ITCs vs. epithionitriles) on the pathogenicity of *A. brassicicola* is greater than the effect of the glucosinolate group from which the glucosinolate-breakdown products were derived.

The *MYB29^OXP^* and *MYB34^OXP^* mutations were expressed against the Ler background and we found no differences between the sensitivities of these transgenic plants, which contain elevated concentrations of aliphatic or indolic glucosinolates, respectively), to *A. brassicicola* and the sensitivity of the Ler wild-type. Taken together with the observation that Ler, like Col-0, contains more aliphatic than indolic glucosinolates [48], this observation lends support to the hypothesis that the nature of the glucosinolate-breakdown products present has a greater effect on resistance/susceptibility to *A. brassicicola* infection than the glucosinolate group from which they were derived, because both of these mutations were expressed against the Ler background, in which nitriles are the more common glucosinolate-breakdown products.

Moreover, we cannot rule out the possibility that the variability observed in the susceptibility of these *Arabidopsis* accessions to *A. brassicicola* may be the result of the diverse nature of these *Arabidopsis* accessions’ immunity and resistance rather than solely a function of the accumulation of camalexin or glucosinolates. Alternatively, Miao and Zentgraf [73] demonstrated that ESP and WRKY53 mediate negative crosstalk between pathogen resistance and senescence. This ESP activity might explain the negative effect of the expression of this protein against the *tgg1/2* background on the pathogenicity of *B. cinerea* isolate B05.10. It might also provide another explanation for the differences we found in Col-0 plants overexpressing ESP, with respect to *A. brassicicola* pathogenicity (**Figure 5**).

The results of our work with the *pen2-2* and *cyp81F2* mutants suggest that indolic glucosinolate-turnover products affect the defense response against *B. cinerea*, but not defense against *A. brassicicola.* These findings are in agreement with recent work involving non-adapted isolates of *P. cucumerina* [34]. A possible mechanism for these effects might involve the ability of *A. brassicicola* to neutralize signaling products, as in the case of the phytoalexin brassinin [58], [74], [75]. Alternatively, these metabolites may not play a role in the defense response against *A. brassicicola* because the fungus is resistant to them or prevents their accumulation by down-regulating the expression of *CYP81F2* and *PEN2* (**Table S1**).

It appears that *A. brassicicola* is a specialist that has adapted to the presence of indolic glucosinolates and prefers their breakdown into epithionitriles, since it less sensitive to these than to ITCs. Although ITCs have been shown to have a larger effect on *A. brassicicola* than epithionitriles *in planta* and *in vitro,* we found that ITC at as low as 10 µM can induce the proliferation of *A. brassicicola*, suggesting that this pathogen can utilize glucosinolate for its own growth (**Figure S5D**). That possibility was also suggested by Giamoustaris *et al.*(1997) [26], who found that an increase in glucosinolate levels enhances the susceptibility of oilseed rape (*Brassica napus* ssp. *oleifera*) to *A. brassicae*.

Overall, we demonstrated that the hydrolysis products of indolic glucosinolates are responsible for the differences observed between plant responses to *B. cinerea* and plant responses to *A. brassicicola.* We suggest that the specialist *A. brassicicola* has adapted to the presence of glucosinolates and their breakdown products and has a preference for nitrile-producing hosts. On the other hand, *B. cinerea* is a generalist that shows no preference for any particular glucosinolate or glucosinolate breakdown group.

An important next step is to examine the effects of glucosinolates on other specialized or broad-host-spectrum necrotrophic fungal pathogens that infect *Brassica* species, to verify that this phenomenon is indeed common to these two groups of fungi, as well as the effects of glucosinolates on adapted or non-adapted pathogens.

## Supporting Information

Figure S1 **Pathogenicity of** ***Botrytis cinerea*** **and** ***Alternaria brassicicola*** **in different** ***Arabidopsis*** **accessions.** *Arabidopsis* leaves from the Col-0, Ler-0 and Ws-0 ecotypes were inoculated with the grape isolate of *B. cinerea*, the B05.10 isolate of *B. cinerea* or *A. brassicicola*. Lesion size was measured 72 h after inoculation with *B. cinerea* and 120 h after inoculation with *A. brassicicola*. Average lesion areas were calculated for 20 to 30 leaves of each genotype and standard errors are presented. Different letters above the columns indicate statistically significant differences at *p*<0.05, according to the Mann-Whitney *U* test.
(TIFF)

Figure S2 **Impact of indole glucosinolate and camalexin on fungal pathogenicity.** *Arabidopsis* leaves from plants with the *cyp79 B2/B3* mutation expressed against the Ws-0 background were inoculated with *B. cinerea* (B05.10 or grape isolate) or *A. brassicicola*. Lesion size was measured 72 h after inoculation with *B. cinerea* and 120 to 192 h after inoculation with *A. brassicicola*. Average sizes of lesions from 30 leaves of each genotype are presented together with the standard error of each average. Asterisks indicate a statistically significant difference relative to the wild- type at *P*<0.01, as indicated by a *t*-test.
(TIFF)

Figure S3 **Impact of the root myrosinase TGG4 on fungal pathogenicity.** *Arabidopsis* leaves from wild-type Col-0 plants and plants with the *35S:TGG4* mutation were inoculated with *A. brassicicola* or *B. cinerea* (B05.10 isolate). Lesion size was measured 120 to 192 h after inoculation with *A. brassicicola* and 72 h after inoculation with *B. cinerea*. Average lesion sizes based on 14 leaves from each genotype are presented together with the standard error of each average.
(TIFF)

Figure S4 **Effect of glucosinolate-breakdown products on** ***Botrytis cinerea B05.10*** **.** Growth of *B. cinerea* mycelia on PDA supplemented with different concentrations of ITCs or solvent only (0.01% methanol). Averages of three to four measurements per treatment are presented together with the standard deviations for each average.
(TIFF)

Figure S5 **Effect of glucosinolate-breakdown products on** ***Alternaria brassicicola*** **.** A-E, Growth of *A. brassicicola* mycelia in PDB supplemented with different concentrations of ITCs or solvent only (0.01% methanol). F, Spore germination and sporulation on PDA plates 24 h post-inoculation. Averages were calculated for three to four measurements per treatment and are presented together with their standard errors.
(TIFF)

Table S1 **Expression pattern of genes involved in tryptophan, GS and camalexin biosynthesis and degradation.**
(DOC)

Data S1 ***In Vitro*** **Growth in the Presence of Isothiocyanates.**
(DOCX)
