# Supplementary figures and images for: The Effects of Glucosinolates and Their Breakdown Products on Necrotrophic Fungi
Source: PLoS One. 2013 Aug 5;8(8):e70771. doi: 10.1371/journal.pone.0070771 (PMC3733641; doi:10.1371/journal.pone.0070771)

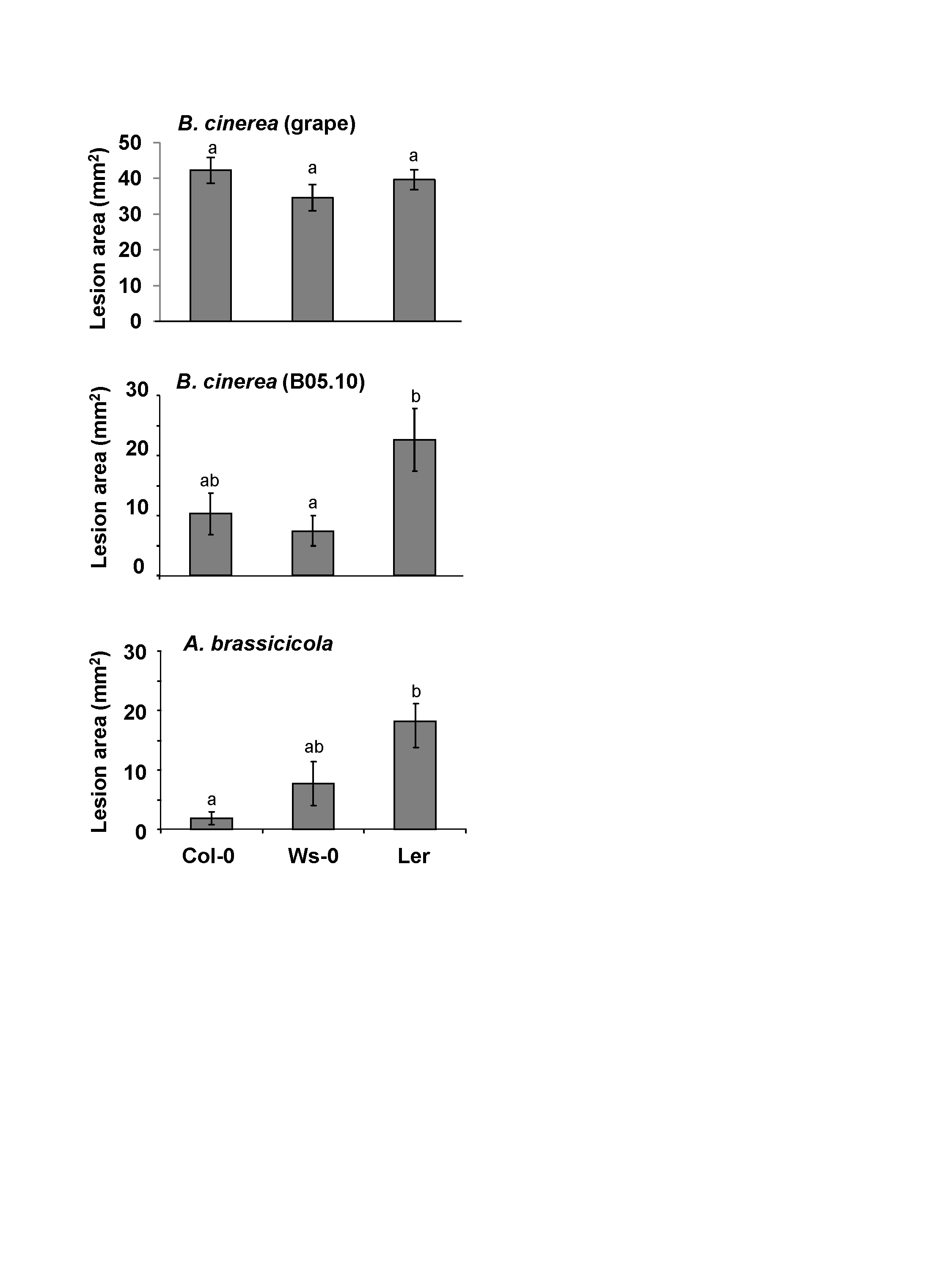

Supplement: Figure S1 — Pathogenicity of Botrytis cinerea and Alternaria brassicicola in different Arabidopsis accessions. Arabidopsis leaves from the Col-0, Ler-0 and Ws-0 ecotypes were inoculated with the grape isolate of B. cinerea, the B05.10 isolate of B. cinerea or A. brassicicola. Lesion size was measured 72 h after inoculation with B. cinerea and 120 h after inoculation with A. brassicicola. Average lesion areas were calculated for 20 to 30 leaves of each genotype and standard errors are presented. Different letters above the columns indicate statistically significant differences at p<0.05, according to the Mann-Whitney U test. (TIFF) [file pone.0070771.s001.tiff]

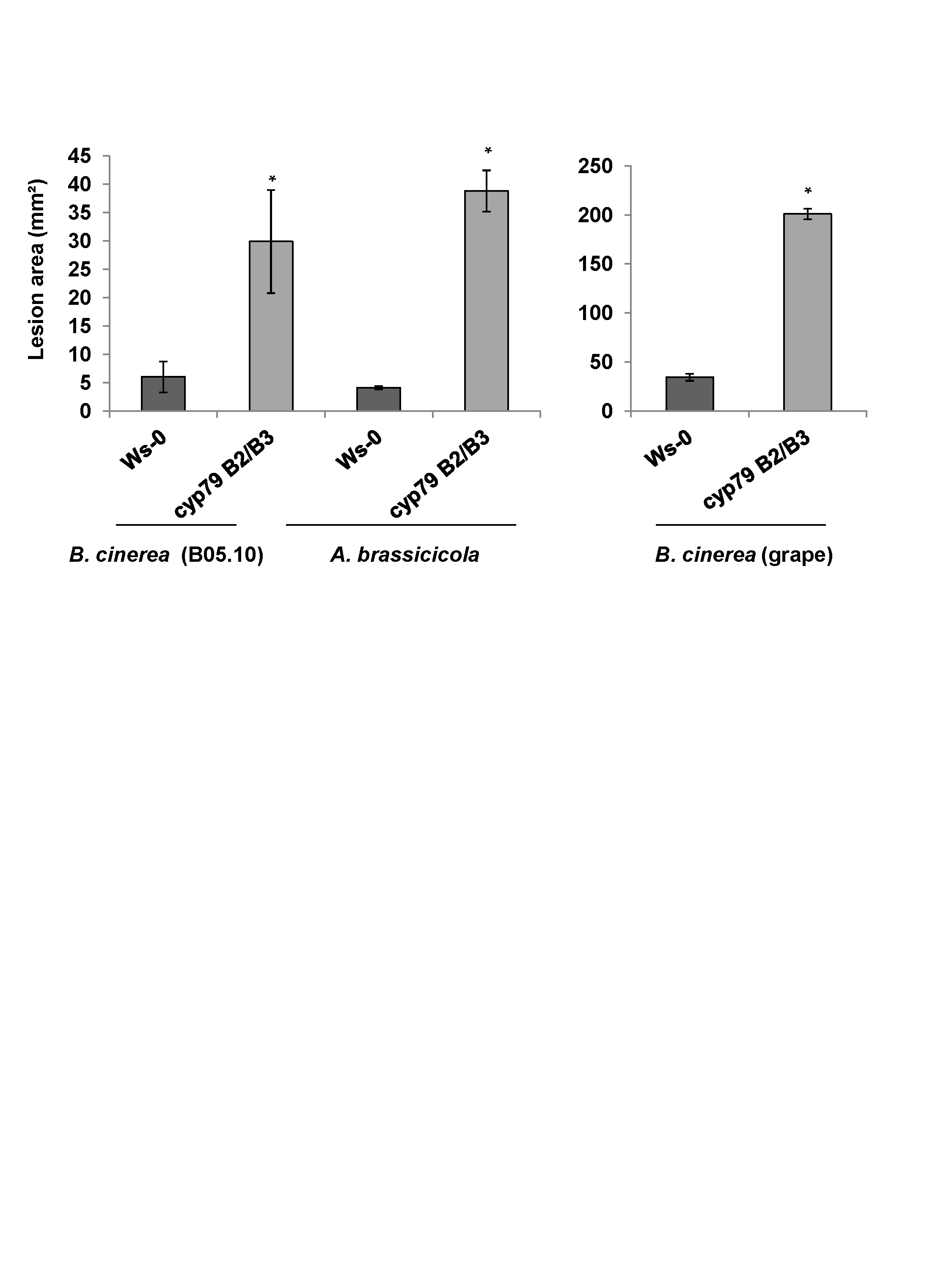

Supplement: Figure S2 — Impact of indole glucosinolate and camalexin on fungal pathogenicity. Arabidopsis leaves from plants with the cyp79 B2/B3 mutation expressed against the Ws-0 background were inoculated with B. cinerea (B05.10 or grape isolate) or A. brassicicola. Lesion size was measured 72 h after inoculation with B. cinerea and 120 to 192 h after inoculation with A. brassicicola. Average sizes of lesions from 30 leaves of each genotype are presented together with the standard error of each average. Asterisks indicate a statistically significant difference relative to the wild- type at P<0.01, as indicated by a t-test. (TIFF) [file pone.0070771.s002.tiff]

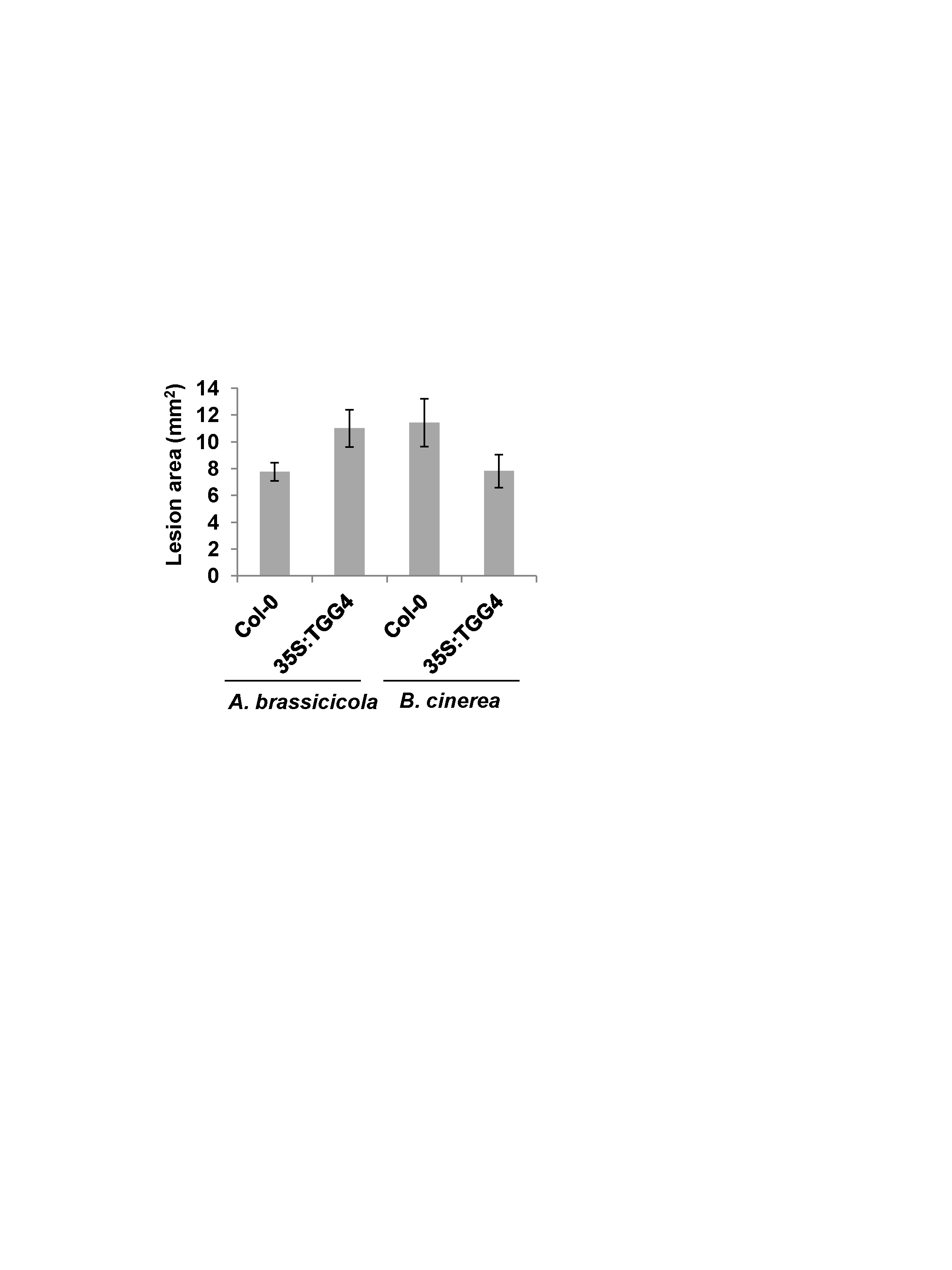

Supplement: Figure S3 — Impact of the root myrosinase TGG4 on fungal pathogenicity. Arabidopsis leaves from wild-type Col-0 plants and plants with the 35S:TGG4 mutation were inoculated with A. brassicicola or B. cinerea (B05.10 isolate). Lesion size was measured 120 to 192 h after inoculation with A. brassicicola and 72 h after inoculation with B. cinerea. Average lesion sizes based on 14 leaves from each genotype are presented together with the standard error of each average. (TIFF) [file pone.0070771.s003.tiff]

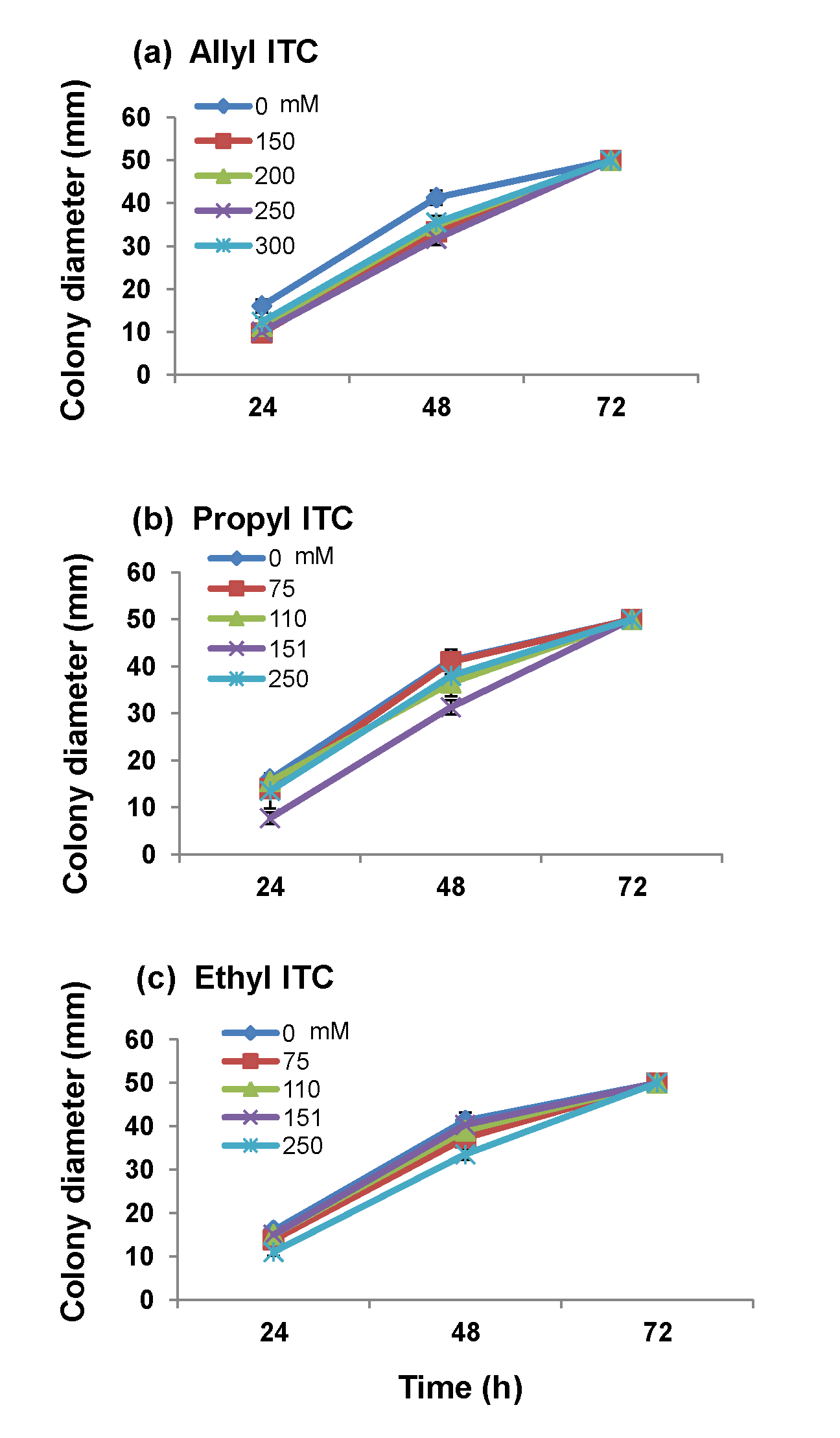

Supplement: Figure S4 — Effect of glucosinolate-breakdown products on Botrytis cinerea B05.10 . Growth of B. cinerea mycelia on PDA supplemented with different concentrations of ITCs or solvent only (0.01% methanol). Averages of three to four measurements per treatment are presented together with the standard deviations for each average. (TIFF) [file pone.0070771.s004.tiff]

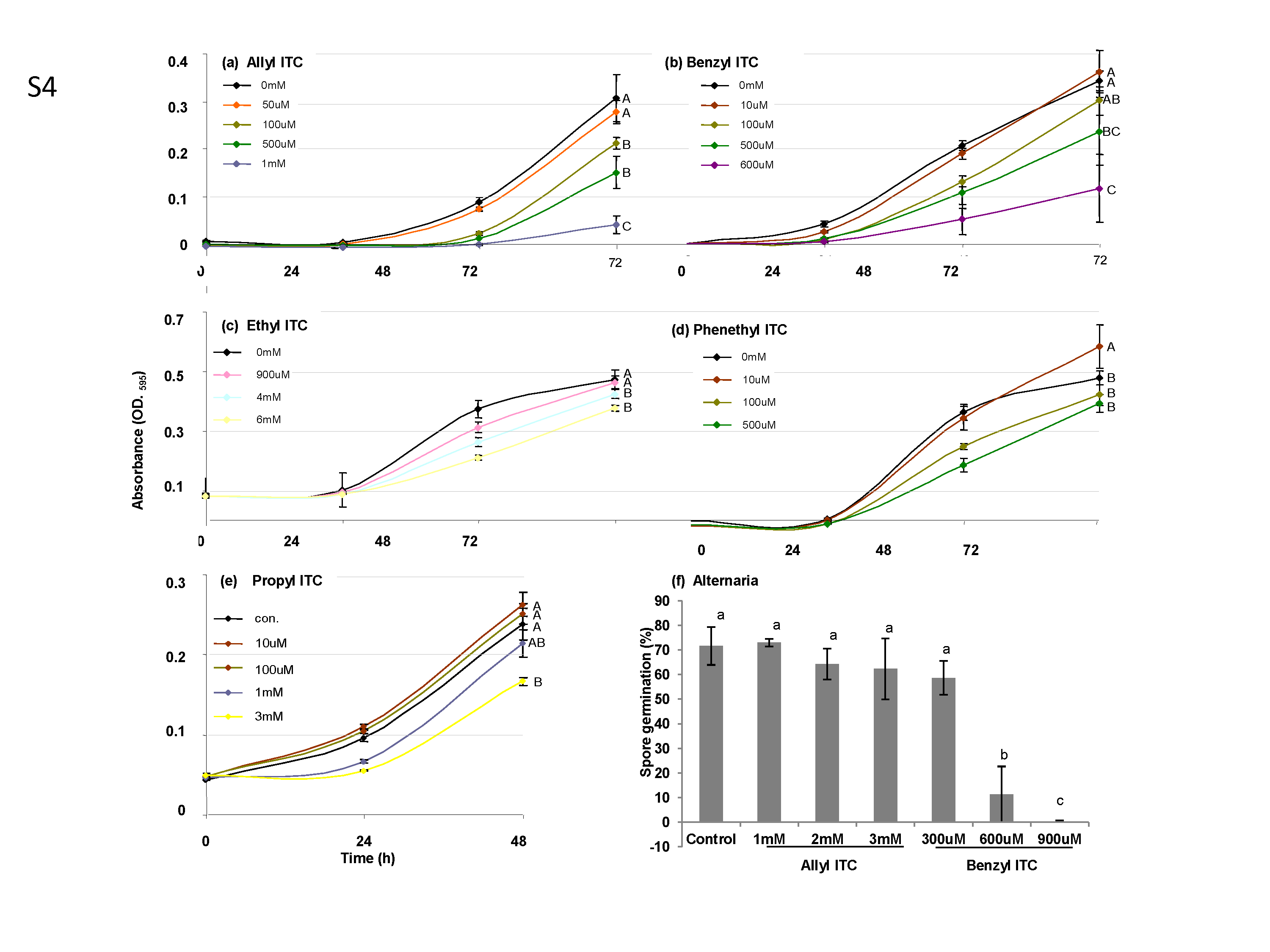

Supplement: Figure S5 — Effect of glucosinolate-breakdown products on Alternaria brassicicola . A-E, Growth of A. brassicicola mycelia in PDB supplemented with different concentrations of ITCs or solvent only (0.01% methanol). F, Spore germination and sporulation on PDA plates 24 h post-inoculation. Averages were calculated for three to four measurements per treatment and are presented together with their standard errors. (TIFF) [file pone.0070771.s005.tiff]
