# Supplementary material for: The Effects of Glucosinolates and Their Breakdown Products on Necrotrophic Fungi
Source: PLoS One. 2013 Aug 5;8(8):e70771. doi: 10.1371/journal.pone.0070771 (PMC3733641; doi:10.1371/journal.pone.0070771)
Supplement: Table S1 — Expression pattern of genes involved in tryptophan, GS and camalexin biosynthesis and degradation. (DOC) [file pone.0070771.s006.doc]

**Table S1** Expression pattern of genes involved in tryptophan, GS and camalexin biosynthesis and degradation*

|  | **Gene** | **Description** | **AGI#** | ***B. cinerea*** | ***A. brassicicola*** | **flg22 1uM 3h** |
| --- | --- | --- | --- | --- | --- | --- |
| ***Tryptophane***  ***biosynthesis*** | *TSB1* | *Tryptophane synthase beta-subunit1* | At5g54810 | 2.47 | 1.50 | 7.68 |
|  | *ASA1* | *Antranylate synthase alpha 1* | At5g05730 | 2.67 | 1.00 | 8.11 |
| ***Methionine***  ***Chain elongation*** | *MAM1* | *methylthioalkylmalate synthase* | At5g23010 | 1.01 | 2.60 | -2.20 |
|  | *MAM3* | *methylthioalkylmalate synthase* | At5g23020 | 2.06 | 3.49 | -1.75 |
|  | *IPMDH1*  *(IMD3)* | *isopropylmalate dehydrogenase* | At1g31180 | 1.03 | 1.69 | -2.24 |
|  | *BCAT2* | *Branched-chain-amino-acid aminotransferase 2* | At1g10070 | 5.41 | -2.01 | 1.56 |
|  | *BCAT3* | *Branched-chain-amino-acid aminotransferase 3* | At3g49680 | -1.19 | 2.07 | -1.64 |
|  | *BCAT4* | *Branched-chain-amino-acid aminotransferase 4* | At3g19710 | 1.24 | 4.00 | -2.44 |
|  | *IPMI1* | *isopropylmalate isomerase 1* | At3g58990 | 1.29 | 3.29 | -3.83 |
|  | *IPMI2* | *isopropylmalate isomerase 2* | At2g43100 | 1.29 | 3.49 | -2.92 |
| ***Aliphatic***  ***Glucosinolate*** | *CYP79F1/F2* | *Cytochrome-P450-79F1/2* | At1g16410/  At1f16400 | 1.5 | 3.61 | -5.88 |
|  | *CYP83A1* | *Cytochrome-P450-83A1* | At4g13770 | 1.28 | 1.64 | 1.49 |
|  | *MYB28* | *Myb transcription factor 28* | At5g61420 | -1.49 | 1.98 | -2.40 |
|  | *MYB29* | *Myb transcription factor 29* | At5g07690 | 1.26 | 3.02 | -1.96 |
| ***Indolic Glucosinolate*** | *CYP83B1* | *Cytochrome-P450-83B1* | At4g31500 | 1.35 | -1.39 | 5.49 |
|  | *CYP79B2* | *Cytochrome-P450-79B2* | At4g39950 | 4.32 | 1.43 | 19.25 |
|  | *CYP79B3* | *Cytochrome-P450-79B3* | At2g22330 | 1.35 | 2.47 | 1.88 |
|  | *MYB51* | *Myb transcription factor 51* | At1g18570 | 1.17 | -2.38 | 18.47 |
|  | *CYP81F2* | *Cytochrome-P450-81F2* | At5g57220 | 1.77 | -3.7 | 788.23 |
|  | *PEN2* | *Penetration2* | At2g44490 | 1.00 | -1.56 | 4.93 |
|  | *MYB34* | *Myb transcription factor 34* | At5g60890 | 1.31 | 1.91 | -6.25 |
| ***Glucosinolate*** | *ST5a* | *Sulfotransferase-5a* | At1g74100 | 1.41 | -1.2 | 4.58 |
|  | *IQD1* | *IQ domain 1* | At3g09710 | -1.02 | 1.11 | 1.09 |
|  | *ESP* | *epithiospecifier* | At1g54040 | 1.17 | 1.2 | -5.55 |
|  | *tgg1/tgg2* | *Myrosinase* | At5g26000/At5g25980 | -1.05 | 2.36 | -1.45 |
| ***Camalexin*** | *CYP71A13* | *Cytochrome-P450-71A13* | At2g30770 | 8.21 | 10.5 | 3.48 |
|  | *Cyp71B15/*  *PAD3* | *Cytochrome-P450-71B15/ Phytoalexin-deficient 3* | At3g26830 | 5.85 | 1.91 | 8.44 |
|  |  |  |  |  |  |  |

*Data represent fold change in gene expression of inoculated/infested plants as compared to uninoculated/infested plants generated from publically available microarray data from Genvestigator database (https://www.genevestigator.com/gv/index.jsp).
