# Supplementary material for: The Effects of Glucosinolates and Their Breakdown Products on Necrotrophic Fungi
Source: PLoS One. 2013 Aug 5;8(8):e70771. doi: 10.1371/journal.pone.0070771 (PMC3733641; doi:10.1371/journal.pone.0070771)
Supplement: Data S1 — In Vitro Growth in the Presence of Isothiocyanates. (DOCX) [file pone.0070771.s007.docx]

Supplementary Material and Methods

For the *in vitro* analysis of growth on media containing ITC, PDA or PDB was supplemented with different concentrations of ITCs (Sigma-Aldrich, St Louis, MO, USA). For the control treatments, methanol (1% v/v final concentration) was added to the growth media instead of ITCs. For *B. cinerea* growth a mycelial plug was placed in the center of a 5- or 9-cm diameter Petri dish and the diameter of the mycelia was measured 24, 48 and 72 h after inoculation. For *A. brassicicola* growth a mycelial plug was placed in PDB and O.D. was measured 24, 48 and 72 h after inoculation.
